# The topology of cyberspace and cybercrime journeys: a framework for analyzing online offender mobility

**DOI:** 10.1186/s40163-026-00294-w

**Published:** 2026-08-20

**Authors:** Asier Moneva, Stijn Ruiter, Wim Bernasco

**Affiliations:** 1 Netherlands Institute for the Study of Crime and Law Enforcement (NSCR), De Boelelaan 1077, 1081 HV Amsterdam, Netherlands; 2 The Hague University of Applied Sciences, The Hague, Netherlands; 3 Vrije Universiteit Amsterdam, Amsterdam, Netherlands; 4 University of Amsterdam, Amsterdam, Netherlands

**Keywords:** Crime analysis, Crime journeys, Crime science, Cybercrime, Cyberspace, Environmental criminology, Offender mobility

## Abstract

**Purpose:**

This paper introduces a novel framework for analyzing online offender mobility.

**Methods:**

Drawing on multidisciplinary insights, we extend two established criminological models for studying offline offending—the geometry of crime and crime journeys—by adapting their core concepts to cyberspace and developing a topology in which the cyber place is defined and becomes the unit of analysis. Just as offenders travel before, during, and after committing crime offline, we argue they also undertake trips before, during, and after committing cybercrime, and that like offline journeys these cybercrime journeys comprise identifiable and measurable components.

**Results:**

We explicitly demonstrate how concepts from the geometry of crime and crime journeys translate from offline to online crime and mobility. The proposed approach enables the systematic formulation of research questions and the measurement of behavioral patterns, facilitating the generation and accumulation of knowledge on cybercrime offending.

**Conclusion:**

The paper illustrates the framework’s theoretical relevance within environmental criminology and its practical application for cybercrime analysis through concrete examples, including an empirical journey into web hacking as a proof-of-concept.

## Introduction

Crime journeys are the sequences of trips that offenders take before, during, and after committing a crime (Bernasco, 2014). These journeys have a set of components (e.g., origin, direction, distance, etc.) that can be leveraged to analyze offender mobility, and its relationship to the crime commission process (Bernasco, 2014; Rengert, 2004). Understanding crime journeys is important for crime analysis as they help to figure out whether, where, when, how, and against what or whom crimes are committed. This, in turn, can inform crime prevention by suggesting how to hinder access to targets, and also crime detection by providing intelligence to investigators (Bernasco, 2014). Crime journeys can also inform crime scripting, an analysis technique that aims to describe sequences of actions that offenders must complete before, during, and after committing a crime and, by so doing, identify possible intervention points (Cornish, 1993; Leclerc, 2017).

Since the first journey-to-crime studies were conceived in the 1970s (for reviews see Rengert, 2004; Rossmo, 2000), crime journeys have been considered geographic by definition, as they focus on how offenders move through physical environments. Research on crime journeys has shown promise in addressing important criminological research questions, such as: “Where in geographical space do offenders start their journey to crime? How do offenders decide on the course of these journeys? And how do they choose the terminus, i.e. the actual criminal target?” (Elffers, 2004, p. 1). While most researchers interested in this topic have focused on the journeys that lead to commit crime (i.e. the journey to crime) (Townsley, 2017), it should be noted that these questions also apply to journeys *from* crime (e.g. Lu, 2003), and to sequences of crime-related trips more broadly. However, questions related to how offenders navigate *online* environments have been largely ignored.

This paper considers cyberspace as a non-geographical place (see Adams, 1998; Batty, 1997).^1^ Our approach is pragmatic rather than ontological; in line with previous conceptual work in criminology (e.g. Miró-Llinares & Johnson, 2018), we argue that retaining the notion of *place* remains conceptually relevant to study cybercrime given its central role in crime theory and analysis. Thinking in terms of places in cyberspace encourages considering online interaction and movement, which is particularly relevant for the concept of journeying, whether in general or in relation to crime. Moreover, the analogy of cyberspace as a place facilitates formulating research questions and deriving hypotheses from existing theoretical frameworks within environmental criminology, thereby providing a solid foundation and contributing to cumulative knowledge.

From this perspective, *cyber* offenders journey to crime, but they do so online, through a cyberspace with spatio-temporal characteristics distinct from those of the geographical space. Some note that distances in cyberspace effectively disappear, since, for example, it is possible to launch a cyber attack from one part of the globe to the opposite in a short time span with minimal effort; and that places appear to be highly volatile, as they can appear online and go offline almost instantly, as if they had never existed (e.g. Grabosky, 2001; Yar, 2005). Given these spatio-temporal differences between online and offline offending, it is important to determine whether the crime journeys framework is also relevant for analyzing the online mobility of offenders, and therefore suitable to answer important research questions in criminology. Specifically, this paper aims to contribute to:

1. Theory development, by examining how and why cyberspace influences offender’ choices and the resulting spatiotemporal cybercrime patterns, as well as proposing a new analytical framework to analyze online offender mobility [regardless of whether the crime is cyber-dependent, cyber-enabled, or cyber-assisted; see McGuire (2020)]; and
2. Its practical application, by defining relevant cybercrime journey components, as well as identifying key criminological questions that this line of research can address (including how these questions differ from those traditionally explored in studies of crime journeys).

The present paper is based on the premise that crime journeys are not entirely free, neither in physical space nor in cyberspace, but that they are constrained by the time available, the architecture of the environment, and resulting crime opportunities that are dependent on the situation (Brantingham & Brantingham, 1993b; Ratcliffe, 2006). Here we are argue that just as geography constrains offenders’ movements and decisions in physical space, the topology of cyberspace similarly shapes and limits cybercrime journeys.

## Towards a geometry of cybercrime based on the topology of cyberspace

Environmental criminology focuses on the situational causes of crime, examining how the immediate environment shapes decision-making and the resulting criminal behavior (Wortley & Townsley, 2017). Within this perspective, the routine activity approach specifies the minimal conditions for crime, emphasizing the convergence of offenders, targets, and opportunities in particular spatio-temporal contexts (Cohen & Felson, 1979). Such convergence produces an uneven distribution of crime events which, over time, gives rise to identifiable crime patterns (Brantingham & Brantingham, 1993a).

As an extension of the above, the Geometry of Crime (Brantingham & Brantingham, 1981) is a framework to identify places in urban settings that are relevant to understand geographical crime patterns. This framework posits that as everyday activities unfold in particular *activity nodes* and the *paths* that connect them, people develop an *activity space*, where they spend most of their time (Brantingham & Brantingham, 1993b, 1995). What people see from their activity space becomes their *awareness space* (Brantingham & Brantingham, 1993a). Although parts of this awareness space may never be physically traversed, they become incorporated into individuals’ mental maps, allowing them to know where these areas are and, within the limits of perception, what they look like. Over time, individuals develop patterns of activity that tend to be regular across daily, weekly, and sometimes even monthly or yearly cycles. Like other people, offenders also have their own activity spaces, where they too develop patterns of activity, both legal and illegal. The activity and awareness spaces of offenders become crime-relevant when they overlap with the spatio-temporal distribution of suitable targets for crime, whether people or objects (Brantingham & Brantingham, 1981). As part of the crime commission process, offenders select their targets in these crime-relevant areas, and—if they become persistent offenders—over time develop mental templates for the sequence of decisions that they make in preparing and committing a crime, including travel and crime site selection (Brantingham & Brantingham, 1978). All these concepts are the building blocks of theories on offender mobility, which crime journey researchers have applied to geographical space. However, to analyze online offender mobility, we first need analogous concepts for cyberspace.

Thinking about cyberspace requires distinguishing between at least two analytically separate but interdependent dimensions produced by computer network technologies: the physical infrastructure and the social interaction layer that operates through it [which is also referred to as the *virtual layer* in social science literature; e.g. Adams (1998); Batty (1997); Dodge and Kitchin (2001)]. The *physical infrastructure* comprises the material and technical components that enable data transmission, including wires, servers, routers, and switches, and the protocol architectures that route packets across networks. These elements are well-defined and fall primarily within the domain of computer science and network engineering (e.g. Kurose & Ross, 2017). By contrast, the *social interaction layer* refers to the digital environment in which humans’ interactions with computers and other humans acquire meaning. It is constituted by websites, online platforms, user interfaces, accounts, and digital services through which individuals communicate, transact, search, offend, and are victimized. This layer operates through the upper layers of network architectures such as the OSI model (Zimmermann, 1980), but is analytically distinct from them: whereas network models describe how data move, the social layer concerns how humans perceive, interpret, and navigate those environments. It is this socially meaningful environment, what we refer to as cyberspace, that generates abstract units of analysis of interest within the geographical and social sciences (e.g. Castells, 2009). Although both dimensions are considered in this paper, the main focus lies on the social interaction layer and the criminological conceptualization of place within it.

For cyberspace topologies to be relevant for cybercrime analysis, in line with the geometry of crime, they must comprise at least the following elements: cyber places,^2^ cyber paths, and cyber spaces.

### Cyber places

For more than three decades, scholars have attempted to conceptualize cyber place with little success, as the concept has not stuck over time or across disciplines. In the 1990s, *geographers* sought to unravel the complex virtual geography of cyber spaces and places (e.g. Adams, 1998; Batty, 1997). In one of the first attempts to establish a typology of cyber places integrating both the digital and physical perspectives of place, Batty (1997, p. 347) distinguishes between digital spaces inside computers, communication spaces between computers, and geographical places altered by digital infrastructure. Batty calls them, respectively, computer spaces or *cspaces*, cyber spaces, and cyber places, and explains that “real place and space are the driving forces, computer or cspace is necessarily a prerequisite to the communications that dominate cyberspace while the very act of use involves infrastructure that is cyber place”. This definition suggests that a cyber place is composed of a combination of various cyber-physical layers, and that the physical structures, facilities, and systems are its main defining element.

Another way geographers have tried to conceptualize places is on the basis of communication. If “a communication system is to communicators what a place is to its inhabitants”, places can be considered virtual topologies in which the nodes are senders or receivers of communication and the edges represent the means of communication between them (Adams, 1998, p. 89). In this case, the dimension of physical layers would lose relevance in favor of the dimension of virtual systems, in which senders and receivers of communication would constitute a networked cyber place. Despite important initial conceptual developments, more than three decades later, definitions from the field of geography remain abstract and difficult to operationalize, hindering further empirical research.

*Computer scientists* view cyber places through a technical lens instead. From this perspective, the physical infrastructure of cyberspace refers to the servers, routers, and switches interconnected to form the infrastructure nodes that host and route information (Kurose & Ross, 2017)—a definition better fitting Adams (1998) conception of virtual place. From a different perspective, early studies on human–computer interaction conceptualized web pages as the nodes around which Internet navigation revolves (Cockburn & Jones, 1996; Tauscher & Greenberg, 1997). In other words, people send and receive communications through cyberspace via networks and through the software contained in computer programs. This more tangible perspective contrasts with that of geographers and allows to move towards an operationalization of cyber place.

For *environmental criminologists*, the concept of place is key. Places play a central role in crime analysis, as they are part of the immediate environment where offenders and their targets converge for crime to occur (Cohen & Felson, 1979; Weisburd et al., 2015). Although internet infrastructure is heavily influenced by geography, the geographical location of a server or data center is rarely relevant to the analysis of the convergence necessary for cybercrime (e.g., Clarke, 2018; Miró-Llinares & Moneva, 2020). Aware of this, some criminologists have developed definitions that incorporate the concept of convergence of offenders and targets online. For example, Brady et al. (2016) [p. 137] argue that “the networks [where information can be retrieved and exchanged through a series of servers] constitute a ‘place’ where motivated offenders can connect or illegally enter into the same network as the potential target”. Miró-Llinares and Johnson (2018, p. 893) defined cyber places as “discrete nodes or areas of activity on the Internet where one is not physically located but can nevertheless act”. Both definitions emphasize the concept of online convergence, yet they are somewhat vague in their references to “networks” and “nodes or areas of activity.” Are all cyber places networks? What exactly are nodes or areas of activity?

To advance scientific research on cyber places, a more precise definition is needed. Building on insights from geographers, computer scientists, and environmental criminologists, we define *cyber place* as:

- A software program—a set of coded instructions that perform specific tasks on a computer—that is:
- Purpose-specific: serves a particular purpose, either by itself or as part of a larger system;
- Interactive: allows communication between people and/or people and computers—either as a sender, receiver, or both;
- Independent: functions on its own but may interact with other software; and
- Encapsulated: has boundaries in terms of functionality, features, and interface.

This definition is based on a set of criteria rather than an analogy to specific technologies, which makes it robust to the emergence of technologies and the development of new cyber spatialities (see Ferreira & Vale, 2021). To illustrate how our definition discriminates, Table 1 classifies, in a motivated manner, 20 examples of digital technologies into cyber places and non-cyber places.

**Table 1:** Examples of cyber places and non-cyber places

Digital technology	Three examples	Is software?	Is purpose-specific?	Is interactive?	Is independent?	Is encapsulated?	Is a cyber place?
Algorithms	Merge Sort, Binary Search, Decision Tree	✓	✓	✓	×	×	No
Cloud storage services	Dropbox, Google Drive, OneDrive	✓	✓	✓	✓	✓	Yes
Command prompts	Windows Command Prompt, Linux terminal, macOS terminal	✓	✓	✓	✓	✓	Yes
Databases	Uniform Crime Reporting Program database, World Bank database, The Human Genome Project database	×	✓	✓	×	×	No
Email services	Gmail, Outlook, Yahoo Mail	✓	✓	✓	✓	✓	Yes
Binary files	.bin files,.jpeg images,.mp3 audio files	✓	✓	×	×	✓	No
Hardware	CPUs, GPUs, hard drives	×	×	×	✓	✓	No
Mobile applications	Whatsapp, Instagram, Uber	✓	✓	✓	✓	✓	Yes
Networks	LAN (Local Area Network), WAN (Wide Area Network), VPN (Virtual Private Network)	×	×	✓	✓	×	No
Online forums	Reddit, Quora, Stack Overflow	✓	✓	✓	✓	✓	Yes
Operating systems	Windows, macOS, Linux	✓	×	✓	✓	✓	No
Plug-ins	SelectorGadget, Wordpress plug-ins, Zotero plug-in	✓	✓	✓	×	✓	No
Programming languages	C, Python, Java	✓	×	✓	×	×	No
Protocols	HTTP (Hypertext Transfer Protocol), FTP (File Transfer Protocol), TCP/IP (Transmission Control Protocol/Internet Protocol)	×	✓	✓	×	×	No
Search engines	Google, Bing, DuckDuckGo	✓	✓	✓	✓	✓	Yes
Social media	Instagram, Bluesky, LinkedIn	✓	✓	✓	✓	✓	Yes
Streaming platforms	Netflix, Spotify, YouTube	✓	✓	✓	✓	✓	Yes
Text processors	Microsoft Word, Google Docs, LibreOffice Writer	✓	✓	✓	✓	✓	Yes
Video games	Fortnite, Call of Duty, Minecraft	✓	✓	✓	✓	✓	Yes
Websites	Wikipedia, Amazon, Internet Archive	✓	✓	✓	✓	✓	Yes

We emphasize that fulfilling the five criteria for a cyber place does not necessarily imply that the place is actually or potentially cybercrime-related. Just as not all geographical places are crime-related, neither are all cyber places. Cyber places unrelated to crime remain relevant for the analysis of online mobility, as they form part of the broader topology of cyberspace. Identifying the places through which an entity moves in a crime journey also requires understanding which places are not part of that route. These may be crime-neutral or even protective environments. We also claim that certain digital technologies, such as databases (i.e. organized data structures), are better understood as objects (e.g. targets) that exist within places (e.g. database management applications), which are themselves hosted on infrastructure (e.g. servers).

### Cyber- activity nodes and paths

The high activity places that host an individual’s routine activities are called activity nodes, and the routes the individual usually travels between activity nodes are called paths (Brantingham & Brantingham, 1993b). Starting from our definition of cyber place, an analogy of these concepts can easily be drawn for cyberspace. For instance, several studies indicate that users have a clear preference for a small set of applications and websites that they visit frequently (e.g. Cockburn & Mckenzie, 2001; De Nadai et al., 2019; Zhao et al., 2014). *Cyber activity nodes* would be these cyber places where a user spends a large proportion of their online time.

Not all cyber places would therefore be cyber activity nodes, but all activity nodes would be cyber places interconnected via computer networks. These networks, sustained by infrastructure components such as servers, facilitate the transfer of information between cyber places—including activity nodes—and enable communication among people, computers, and between people and computers. Networks are the streets and highways of cyberspace acting as *cyber paths*. These cyber paths connect cyber places and infrastructure nodes and enable travel between them, taking two distinct forms: in the physical infrastructure, they include wired and wireless connections like cables and satellite communication, while in the social interaction layer, they manifest as user actions like clicking hyperlinks or pressing keys. For example, a classic study referred to ‘paths’ as the sequences of website visits (Catledge & Pitkow, 1995). The way individuals navigate from one cyber place to another via hyperlinks, and how the information they transmit travels through the physical infrastructure, together define their cyber journeys.

### Cyber spaces

In geographical space, activity spaces are a combination of activity nodes and paths (Brantingham & Brantingham, 1993b). However, unlike in geographic space, in cyberspace people do not spend time on the paths, but wait in one cyber place until the information they want to communicate is transmitted through the path and back. Since paths do not create additional ‘space’ where one can spend time on, their function is to connect certain cyber places in a sequential manner. It is possible for individuals to use tools such as browsers, VPNs, and proxies to deliberately route their traffic through specific physical networks while avoiding others. However, regardless of the complexity or length of the chosen path, the connected cyber places remain the same. Therefore, *cyber activity spaces* can be defined as the most common sequences of cyber activity nodes, and will vary between individuals depending on their activity patterns.

What people see from the activity spaces is their awareness space (Brantingham & Brantingham, 1993a).^3^ It is these areas in which offenders are most likely to look for their targets. In cyberspace, however, *cyber awareness spaces* have an important particularity compared to geographic ones, namely that it is not possible to look beyond the cyber place in which one is located. Cyber places are encapsulated, and unlike geographical paths, cyber paths do not allow to see through them. And although it is possible to see certain signs pointing to other cyber places (hyperlinks, hyperlinked action buttons), it is not possible to become familiar with that place until one visits it. This means that in cyberspace there is a perfect overlap between the cyber activity space and the awareness space of an individual.

## Transdisciplinary insights into online mobility

Since the 1990s, studies in fields such as information security, computer networking, and human–computer interaction have analyzed human behavior in cyberspace to shed light on online decision-making processes, including how users move between different activity nodes.

In the early stages of this field, a classic study used client-side log files to examine the browsing strategies of university staff and students (Catledge & Pitkow, 1995). The authors described the mean time between events, the length of the paths between websites, and characterized the websites based on these path lengths and the frequency of access. They also described a “spoke and hub” structure in navigation patterns (Catledge & Pitkow, 1995, p. 1070), in which users primarily operated by continuously moving back and forth within the same web domains. Shortly afterward, another study introduced a graphical software tool that allowed researchers to visualize web navigation as a network, where nodes represent websites and edges represent the traversals between pages (Cockburn & Jones, 1996). Others supported this two-dimensional representation of web browsing and argued that, consistent with Catledge and Pitkow (1995) findings, web browsing was rarely linear. Instead, much of the traffic flowed through a few hubs that acted as “branching points” (Tauscher & Greenberg, 1997, p. 101). The disproportionate attention certain web nodes receive can be explained by a pattern of repeat visits or *revisits*, defined as “navigating to a previously visited page” (Cockburn & Mckenzie, 2001, p. 909). Studies from the late 1990s and early 2000s estimated revisit rates for certain nodes ranging from 81% (Cockburn & Mckenzie, 2001), to 61% (Catledge & Pitkow, 1995), and 58% (Tauscher & Greenberg, 1997) reported in other studies. These figures indicate that users have a clear preference for a small set of frequently visited nodes along with their nearby cyber places.

Subsequent studies in these and other fields expanded these insights further. Another classic study recorded the online activities of eight participants from a university community as they browsed the Internet as part of their daily routines (Byrne, John, Wehrle, & Crow, 1999). The authors observed that the most frequent behaviors involved navigating the Internet—moving from one node to another—and configuring web browsers, while the most time was spent using the information found. Another study modeled the searching behavior of 12 Internet experts, finding that greater expertise was associated with more successful search outcomes (Hölscher & Strube, 2000). Similarly, Cockburn and Mckenzie (2001) analyzed the search histories of 17 participants and found they visited an average of 42 websites daily—double or even triple the figures reported in earlier studies—highlighting the growing prominence of online activity. Their search queries were classified into three categories according to user intent: navigational, aimed at reaching a specific node; informational, aimed at retrieving information from a node, and transactional, aimed at reaching a node to find another (Broder, 2002). The navigational type appeared to be the least frequent of the three, yet it allowed for more direct and therefore smoother internet navigation. Another study recorded the activity of 55 members of the university community as they completed specific online purchasing tasks (Karimi et al., 2015). Based on whether participants had little or extensive substantive knowledge about the task, and whether their decision-making style was more or less conservative, the authors identified four types of consumers that displayed distinct behavior. These studies indicate the existence of behavioral patterns in users’ online decision-making processes as they navigate cyberspace.

More recently, several studies on human (cyber) dynamics have leveraged digital trace data and location-tracking technologies to examine patterns of online and offline exploration and return behaviors, highlighting the similarities and differences between these two domains. For example, visits to websites and physical locations may be explained by two behaviors: as users visit more sites, they become less likely to explore new ones, and they tend to return more often to sites they visited early (Zhao et al., 2014). Similarly, geographic mobility and app usage over six months show that just as people spend most of their time in a few physical locations, mobile phone time is similarly concentrated in a few apps, with exploration of new apps happening more slowly (De Nadai et al., 2019). Online exploration also appears to decay over time in a forum (Reddit), with prior mobility explaining 67% of the variance in the intention to explore new communities (Hu, Xia, & Luo, 2019), confirming that online behavior is more repetitive than offline. Another study shows that browsing routines are highly predictable, and that users spend an average of 30 seconds on each domain visit (Kulshrestha et al., 2021). These patterns of repetitive behavior and infrequent exploration have also been observed in TV viewing and online shopping data, demonstrating that people mostly revisit familiar sites and only occasionally try something new (Wang et al., 2023). These studies support the idea of some online nodes as key points in digital navigation and highlight the consistent ways in which online and offline mobility patterns align.

Collectively, prior research summarized above suggests that empirical regularities observed in offline mobility may also apply to online mobility, albeit with some differences in magnitude. In particular, it appears that: (1) online behavior is more spatio-temporally concentrated than offline behavior; (2) awareness space expands more slowly online than offline; (3) past online and offline mobility strongly predicts future mobility; (4) location exploration rates decline over time more rapidly online than offline; and (5) users exhibit preferential return to familiar online and offline nodes. Environmental criminology rests on propositions of a similar nature (Wortley & Townsley, 2017), but so far, no framework has been proposed to systematically observe whether these or other propositions also hold in online environments—and, if so, how they could be measured and what important criminological questions they might help us answer. Before reaching that point though, and just as was done in the development of crime pattern theory within environmental criminology (Brantingham & Brantingham, 1981), it is first necessary to articulate the geometry of cybercrime and how it aligns with the spatio-temporal particularities of cyberspace.

## A framework to analyze cybercrime journeys

To study offender mobility in geographic space, criminologists developed the concept of crime journeys. According to Bernasco (2014), a crime journey is the departure (and sometimes return) tour taken by an offender from a relevant reference location, which in turn refers to “the (main) home of the individual or any other the location from which the individual regularly departs and returns to on a daily basis” (p. 2). In cyberspace, individuals journey through the social interaction layer. Through their screens, users exercise control over their actions, such as choosing an application or clicking a specific link to visit a webpage. From a criminological and behavioral perspective, examining this process allows to analyze the decision-making process of offenders through their online journey. And just like understanding the geography is essential to analyzing offline mobility, understanding the topology of cyberspace—cyber places, activity nodes, paths, and spaces—is key to analyzing online mobility.

Drawing on the concept of crime journeys and taking into consideration the topology of cyberspace, we propose a framework for analyzing online offender mobility: the *cybercrime journeys*. Journeying implies movement; in this case, online movement. A user visits a cyber place when it is open (the software is running), and focuses their attention on it. We then define movement as the transition from one cyber place to another through the shifting of the user's attention. A cybercrime journey therefore encompasses the sequence of nodes that an individual traverse across the social interaction layer of cyberspace before, during, and after committing a crime.

**Fig. 1:**
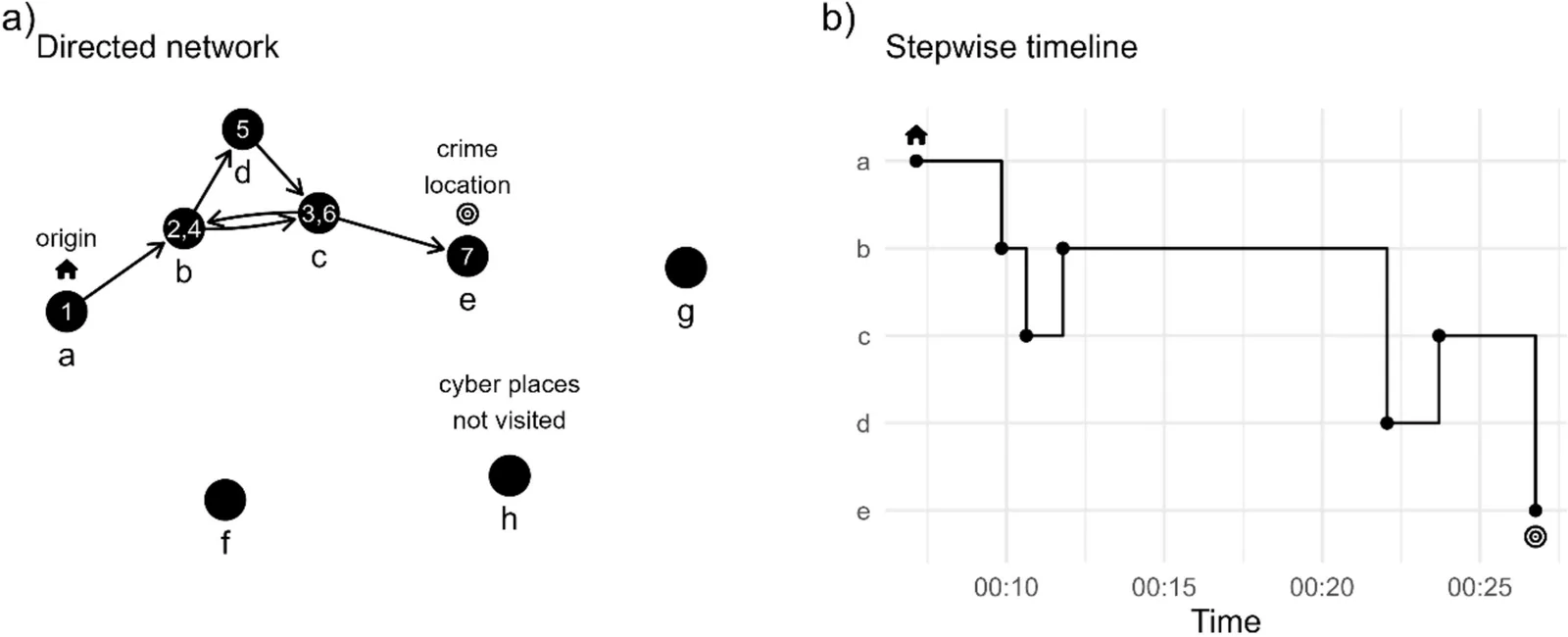
Schematic representation of a cybercrime journey as: **a** a directed network, and **b** a stepwise timeline. In panel (a), Hindu-Arabic numerals within nodes indicate the sequence of cyber places visited by the user

A key part of crime analysis is data visualization. In this section, we present two ways to represent cybercrime journeys (Fig. 1): as a directed network (Panel a), and as a stepwise timeline (Panel b). Networks are a common way to represent topological relationships, especially in communication systems (see Dodge & Kitchin, 2001). They structure interactions between entities like devices and users. Given the aforementioned topology of cyberspace, we propose representing cybercrime journeys as directed networks where nodes represent places, and edges—depicted as arrows between consecutively visited places—capture sequences of visits across places. By following the arrows, journeys can be reconstructed step by step. The disconnected nodes represent cyber places that are part of the subject’s activity space, but not part of the journey. This approach also enables the use of network properties for both visualization and statistical analysis. Alternatively, or as a complement, we represent cybercrime journeys as stepwise timelines. Here, the cyber places visited are arranged along the y-axis, and the sequence of visits is shown by a line connecting them over time. The added value of this visualization is that it captures the temporal dimension of the journeys, indicating how long individuals spend at each location.^4^

In both cases, cybercrime journeys can be represented at either an individual and aggregate level. Individual visualizations allow for detailed analysis of specific cases, while aggregate visualizations reveal broader activity patterns across multiple journeys.

Crime journeys can be dissected into components for analysis. Building on the components proposed by Bernasco (2014), in Table 2 we propose an adapted version that considers the unique topology of cyberspace. This adaptation provides analogies for each original component that, in our view, remains relevant to cybercrime journeys.

**Table 2:** Components of crime journeys and their analogues in cyberspace

Component	Traditional definition	Cyber definition/analogy	Question it answers
Motivations	The reason that inspires an individual to start traveling	Same. E.g. to hack a website	Why does the individual travel?
Origins	The reference location	The cyber place serving as the usual entry point to cyberspace. E.g. a web browser	Where does the individual start?
Destinations	The locations where an individual interrupts travel to perform an activity. It can also be the ‘origin’ in return trips	Same, replacing locations with cyber places. E.g. a search engine	Where does the individual intend to arrive?
Routes	The sequence of locations that an individual visits between the ‘origin’ and the ‘destination’	Same, with locations as cyber places. E.g. web browser → search engine → exploit database → target website	What route does the individual take?
Times	The moment when an individual departs from the ‘origin’ and arrives at the ‘destination’	Same. E.g. from 00:00 to 01:00 h	When does travel occur?
Durations	The time that an individual takes to travel the ‘route’. It can also refer to ‘route’ sections	Same. E.g. one hour	How long does travel last?
Speeds	The speed at which an individual travels along the ‘route’	The average number of cyber places visited per unit of time. E.g. 10 cyber places per minute	How fast does travel happen?
Distances	The total distance that an individual covers along the ‘route’	The number of paths traveled between cyber places, including repeats. E.g. 10 paths	How far does the individual travel?
Co-travelers	The sequence and timing of any company that an individual has along the ‘route’	The sequence and timing of users communicating with an individual during the journey. E.g. a forum user, another hacker	With whom does the individual travel?
Crime locations	The location of the target of the crime	Same. E.g. the target website	Where does the crime take place?

## Empirical illustration of the framework

To demonstrate the feasibility of the proposed framework, this section presents a proof of concept based on data from a pilot study by Moneva et al. (2024) on criminal expertise and hacking efficiency. The data, collected in a computer lab in 2021, consist of monitoring software logs recording a web hack conducted by an expert hacker, which contains information about trips made before, and during the hack was committed. This hack can be described as a premeditated human journey to crime with a fixed destination on a vulnerable target website. The setup predetermined some of the exploitation techniques used to gain access to the system and therefore influenced how the journey unfolded. Because the monitoring software did not capture any activity within the virtual machine running a Kali Linux operating system—containing many hacking tools—part of the journey is missing. While incompleteness limits its use for theory testing, it does not compromise its value for empirically illustrating the current framework, as it shows how offenders may move within the topology of cyberspace. Since this section is only intended to serve as an example of how the framework can be applied, rather than providing a comprehensive empirical analysis, we refer readers to the original open-access publication for a detailed description of the methodology.

**Fig. 2:**
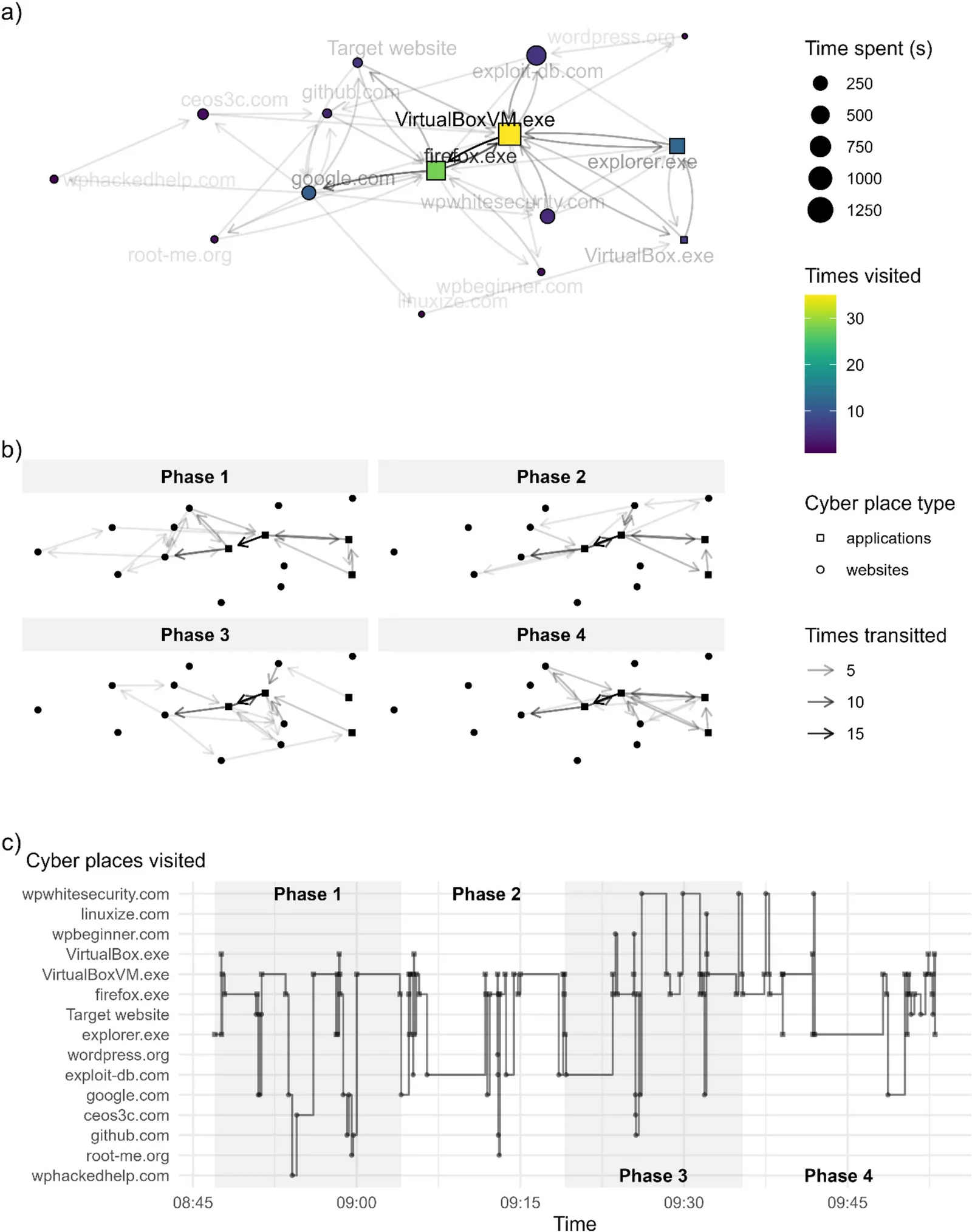
Multiple representations of a journey to web hacking. **a** The journey as a network, with activity nodes and paths highlighted according to visit and transition frequency, and time spent. **b** Four sequential snapshots of the network to depict the journey’s progression over time. **c** The step-by-step journey across time and cyber place

Figure 2 illustrates the hacker’s journey into web hacking across space and time in three distinct ways. We draw on the relevant components of cybercrime journeys to describe the hack. Panel (a) maps 15 unique destinations visited by the hacker into a network, represented as either web (circles) or native applications (squares). The network reveals uneven patterns in both time spent and transitions between locations. These are visualized through node size and edge color, with larger nodes and darker edges indicating more intensive activity and frequent movement. Panel (b) breaks down the hacker’s route into four phases, while Panel (c) provides a step-by-step reconstruction of the journey. The latter panel shows that the activity originated on the File Explorer (explorer.exe) and lasted approximately 66 min. The length of individual visits to cyber places ranged from 1 second to over 6 minutes. On average, the hacker traveled at a speed of 4.4 cyber places per minute, traversing a total of 122 paths. It is known that the hacker traveled alone, and that the crime—a defacement—was committed in the target website.

This example illustrates the feasibility of the framework, and shows some basic descriptive insights that cybercrime journeys can systematically provide. However, in practice, some journey components may be difficult to observe. To facilitate research on cybercrime journeys, next section discusses types of data required, how they can be collected, and the specific insights they would provide.

## Data sources to analyze cybercrime journeys

Several methodologies have been used to reconstruct crime-related, spatio-temporal activity patterns. To do so, researchers often rely on self-reported data, such as diaries, space–time budget methods, and mobile applications (for reviews see Hoeben et al., 2014; Solymosi et al., 2021). While insightful, such methods are susceptible to measurement error, including recall and social desirability biases, which can affect the validity and reliability of the data. On rare occasions, researchers collect objective measures using GPS trackers and cell tower location data (Griffiths et al., 2017; Rossmo, Lu, & Fang, 2011), which avoid the aforementioned biases, but often lack contextual details about offenders’ activities and motivations.

Since empirical studies on offender mobility in cyberspace are scarce, studies on legal online activity can illustrate the types of data that could be leveraged to analyse cybercrime journeys. As with offline activity, self-reported online activity data face limitations (for a meta-analysis, see Parry et al., 2021). Objective measures are therefore preferred for a more accurate account of online activity. Monitoring software, for instance, can provide detailed data on users’ spatio-temporal activity by capturing browsing history (Kulshrestha et al., 2021; Zhao et al., 2014) and application usage (De Nadai et al., 2019). Applied to criminological research, these methods can offer valuable insights into online offender mobility. Other data types, such as screen recordings and time-stamped keystrokes, have already been used to examine online offender activity in controlled settings, allowing highly reliable analyses of cybercrime journeys.

These data collection efforts may be part of research designs that involve organizing activities such as hackathons with consenting participants, and developing honeypot infrastructures involving subjects who were not asked for consent. For example, researchers developed a realistic cybercrime challenge for penetration testing and software engineering students, presented as a monitored capture-the-flag exercise (Moneva et al., 2024). Another research team designed a honeypot simulating a cybercrime market, in which users from other online criminal markets could test fake compromised machines under video monitoring (Ricaldi et al., 2025). Whereas laboratory designs offer greater control over the study setting, honeypot designs have the advantage of enabling the application of the framework in ecologically valid settings. In the spirit of crime science (Clarke, 2010), such research designs typically require more preparation than surveys or interviews, as they often involve interdisciplinary work and collaboration with third parties.

There are many data types and sources that allow researchers to observe cybercrime journeys, either partially or completely. The observable components of a journey depend on the capabilities of the data collection tool. Some software can simultaneously capture multiple data types (e.g. monitoring software may collect screen recordings, system event logs, and keystroke logs), and various techniques exist for capturing the same data type (e.g., web browsing can be recorded through a browser’s history or via packet capture of HTTP traffic). Without aiming to be exhaustive, Table 3 provides an overview of some of the sources for collecting relevant data to analyze cybercrime journeys.

**Table 3:** Cybercrime journey data sources and journey components captured

Source	Data type	Components potentially captured
Screen recordings	Video of user interaction and interface navigation	Origins, destinations, routes, times, durations, speeds, distances, co-travelers, crime locations
Surveys and interviews	Qualitative self-reports of activity and motivation	Motivations, origins, destinations, routes, times, durations, co-travelers, crime locations
Police files and court sentencing records	Qualitative reports of police and judicial investigations	Motivations, origins, destinations, routes, times, durations, co-travelers, crime locations
System event logs	OS-level events (e.g. log-ins, process starts, file access)	Origins, destinations, routes, times, durations, speeds, crime locations
Packet capture	Network traffic	Origins, destinations, routes, times, durations, speeds, distances
Application logs	Specific application usage	Destinations, routes, times, durations, speeds, crime locations
Web browsing history	Sequence of URLs visited	Origins, destinations, routes, times, durations, speeds
Web scraping and OSINT	Digital footprint within or across cyber places	Destinations, routes, durations, co-travelers, crime locations
Keystroke logs	User input at cyber places (e.g. keystrokes, commands, searches)	Motivations, co-travelers
Detection systems	Security logs (e.g. alerts, incidents) and associated metadata	Crime locations

Some additional considerations for data analysis are important. Most of the sources listed—except for surveys and interviews—provide objective measures. Some sources, such as screen recordings, capture numerous components but pose considerable challenges for data analysis, as parsing video data into a structured tabular format often requires manual extraction that is both time-consuming and costly. Conversely, sources like surveys and interviews provide a substantial amount of journey components and have the advantage of being more accessible for both data collection and analysis. While some components (e.g., destinations, routes, durations) can be captured in multiple ways, others (e.g. motivations and co-offenders) require specific data collection tools. Therefore, the choice of data source should be carefully considered in light of the research question, as well as resource constraints and the challenges involved in data analysis.

It should also be noted that there are other data sources that provide valuable, albeit incomplete, information for understanding cybercrime journeys. For example, while application logs and browsing history capture critical segments of the human journey, they are insufficient on their own to represent complete journeys. Nevertheless, browsing data can be particularly insightful for examining preparatory behaviors within the crime-commission process (i.e. reconnaissance). These web nodes could be complemented by application nodes to capture the two most prevalent types of cyber places within the human journeys. Additional data sources excluded from the table, such as application programming interfaces and web analytics, enable tracking behavior within a single cyber place and can be useful for understanding micro-patterns of activity within segments of the journey. In any case, it is important to understand what can be derived from each data source and for what purpose.

## Purpose of researching cybercrime journeys

The precise definition of the basic elements of the topology of cyberspace, along with the operationalization of journey components, facilitates the formulation of testable hypotheses about where, when, and how cybercrimes are committed, illustrating the framework’s potential for theory building and practical application. For example, it becomes possible to draw on established hypotheses about crime location choice and offender mobility in geographical space, replace geographic concepts with their topographic analogues, and thus derive new hypotheses for online offending.

### Theory development using the topology of cyberspace

Empirical research using *the topology of cyberspace* can shed light on how and why the immediate cyber environment influences criminal behavior and how cybercrimes show spatiotemporal patterns, offering key insights for environmental criminology and crime analysis (Wortley & Townsley, 2017).

An established empirical regularity in criminology is the concentration of crime in specific places (Sherman et al., 1989; Weisburd, 2015). Two main explanations for this phenomenon are known as the ‘flag’ and the ‘boost’ accounts (Pease, 1998). The *flag* account argues that certain places possess characteristics that make them prone to crime, either by attracting offenders or by lacking effective crime control mechanisms. In contrast, the *boost* account suggests that during an initial attack, offenders either discover or create vulnerabilities that increase the target’s attractiveness for repeat offenses. In cyberspace, applications, websites, networks, and servers may similarly be vulnerable to attacks or perceived as particularly valuable by offenders, making them more likely to be targeted and to experience concentrations of crime. This assumption leads to the following hypotheses:

#### H_1_

 Cybercrime is disproportionately concentrated in a few cyber places.

#### H_1.1_

 Cybercrime is more likely to occur in cyber places that are perceived as more valuable or vulnerable.

#### H_1.2_

 Cybercrime is more likely to occur in cyber places that have been victimized.

At the individual level, one explanation for certain crime patterns is that individuals are more likely to commit offenses within their activity and awareness spaces (Brantingham & Brantingham, 1978, 2017). Given that in cyberspace the awareness space overlaps with the activity space (see “Cyber spaces” section), the hypothesis would be:

#### H_2_

 Offenders are more likely to attack targets at cyber places within their cyber activity space.

In line with the least effort principle (Zipf, 1949), research on the journey to crime has shown that offenders tend to prefer crime trips requiring the least travel effort (Bernasco & Nieuwbeerta, 2005). Although navigating cyberspace demands less effort than moving through physical space, it still involves cognitive and procedural effort. The following hypotheses follow:

#### H_3_

 Offenders are more likely to attack targets at cyber places that are topologically close to their cyber activity space.

#### H_3.1_

 Offenders are more likely to attack targets at cyber places topologically close to their origin than targets topologically distant from their origin.

#### H_3.2_

 Offenders are more likely to attack targets at cyber places topologically close to their prior targets than targets topologically distant from their prior targets.

Consistent with the boost account for repeat victimization, research suggests that the commission of a crime provides offenders with unique knowledge of the crime location and the targets within it, increasing the likelihood that the location will be selected for a repeat offense (Bernasco, 2008; Farrell et al., 1995). This likelihood appears to increase when the prior offense is recent and when the number of prior offenses at that location is higher (Lammers et al., 2015). However, the limited existing evidence on similar patterns in cybercrime suggests that this effect may be less pronounced in cyber places. Data on self-reported website defacements indicate that while a small group of prolific offenders commit most repeat attacks, only a minority of offenders repeatedly target the same websites (Moneva et al., 2022). However, given the limitations of the data analyzed, the evidence to contest the original claim remains insufficient. To test this prediction further, one could hypothesize:

#### H_4_

 Offenders are more likely to repeatedly attack targets at the same cyber places.

#### H_4.1_

 The more recently offenders have targeted a cyber place, the more likely they are to target the same cyber place again.

#### H_4.2_

 The more frequently offenders have targeted a cyber place, the more likely they are to target the same cyber place again.

### Practical application using cybercrime journeys

Beyond providing a framework for theory development, *cybercrime journeys* also enable the systematic generation of research questions and answers concerning online offender mobility. Empirical findings can then be organized around journey components, producing standardized and comparable measures across studies, which in turn supports the cumulative generation of knowledge.

Traditional research on journey-to-crime has primarily focused on the distances that offenders travel to commit the crime, and on identifying the places where crime is most likely to occur (Townsley, 2017). Other relevant questions concern the origins of these journeys (Rossmo, 2000), and the related decision-making processes, including crime location choice (Bernasco, 2014; Elffers, 2004). These questions would also be relevant for the study of cybercrime journeys, as they provide insight into how offenders manage to converge with attractive targets across cyberspace and time. The resulting knowledge may help identify points of intervention to block or disrupt cybercrime journeys. However, just as it is necessary to rethink the geometry of crime to adapt it to the digital environment through a topology of cyberspace, it is also essential to reconsider how certain aspects related to crime commission change in the case of cybercrime. For example, while distances and effort continue to shape online offender decision-making, their nature and likely impact are different and arguably less pronounced in cyberspace.

Without aiming to be exhaustive, we outline here some of the questions that could be addressed using the cybercrime journeys framework and that are relevant for the prevention and investigation of cybercrime.

RQ_1_: From which cyber places do offenders typically begin their journey-to-cybercrime?
RQ_2_: What routes do offenders follow to commit specific forms of cybercrime, and how do these differ from the routes used to commit other cyber offenses?
RQ_3_: What journeys do offenders make when they are not committing cybercrime but are still learning about cyberspace?
RQ_4_: What types of information do offenders transfer and receive within the cyber places they visit during their journeys?
RQ_5_: Which servers and networks host the cybercriminal tools and services that facilitate crime commission?
RQ_6_: How does communication between individuals navigating cyberspace occur during their journeys?
RQ_7_: How do offenders select their target relative to the initiation of their journey-to-cybercrime?

The journey components would serve to operationalize the key concepts of these questions. Origins, routes, data types, destinations, co-travelers, and crime locations are among the aspects that, in these cases, would enable the systematic measurement and contextualization of corresponding empirical observations.

## Discussion

This article introduced a novel framework for analyzing online offender mobility by adapting the geometry of crime (Brantingham & Brantingham, 1981) to a topology of cyberspace. This lays the foundations for subsequent theoretical development, with the cyber place as the unit of analysis. Defining cyber place, in turn, enables the operationalization of place-related concepts in cyberspace—such as online offender convergence settings, cybercrime generators, and cybercrime attractors (Brantingham & Brantingham, 1995; for the original concepts, see Felson, 2003)—and the systematic analysis of cybercrime within them. Under this definition, digital platforms and ecosystems can be understood as collections of connected digital environments where interactions occur, and thus as networks of interrelated cyber places, and microplaces within them. For example, some studies have used the term virtual or online offender convergence setting ad hoc to describe underground forums or instant messaging services where potential offenders meet and exchange information (e.g. Garkava et al., 2024; Soudijn & Zegers, 2012). Similarly, social media platforms can serve as cybercrime generators for social offenses such as harassment, hate speech, and disinformation, while database management systems containing sensitive data may function as cybercrime attractors for financially motivated offenses, including data theft, phishing, and ransomware. Similarly, the framework enables the evaluation of place-based interventions, such as hot-spot policing, as well as related phenomena including crime displacement and spatial patterns of near-repeat victimization. By formally defining cyber place, our framework moves beyond ad hoc usages, enabling the systematic identification, classification, and analysis of cybercrime places within the topology of cyberspace.

We then extended the concept of crime journeys (Bernasco, 2014) to cyberspace. This expanded framework identifies both analogous and unique components of cyber journeys, raises new research questions, and enables systematic observation of the cybercrime commission process, which may be organized into new propositions, facilitating the development of new hypotheses and theories of cybercrime offending. At the same time, analyzing cybercrime journeys offers valuable insights for both the prevention and investigation of cybercrime. For instance, distinct spatiotemporal patterns in offender journeys can support *disruption* by revealing key cyber places suitable for intervention, and *detection* by uncovering consistent modus operandi among individuals or groups.

The framework presented here is applicable to the study of any form of cybercrime, whether cyber-dependent, cyber-enabled, or cyber-assisted (see McGuire, 2020). In the empirical illustration, we demonstrated its application to a form of hacking—often considered the archetypal cybercrime. However, the framework could equally be applied to offenses such as online fraud, cyber stalking, or the spread of disinformation. The key premise is that whenever offenders move from one cyber place to another as part of the crime commission process, their online movements can be systematically mapped. This includes activities such as preparing and distributing deceptive phishing emails, gathering open-source intelligence, or posting and amplifying content on social media platforms. We therefore expect different crime types to produce distinct journeys, revealing offense-specific patterns of mobility in cyberspace.

A challenge in analyzing online mobility is that, unlike the offline world, offenders can be in more than one cyber place at a time. For example, a user can open multiple applications simultaneously on one device, or a single application across multiple devices. Given the contraction of distances in cyberspace (Yar, 2005), having multiple cyber places open at once can be interpreted as having several places quickly accessible. In this case, it is important to identify where the user’s attention is focused, such as by identifying which application is active or where the cursor is located. This helps determine in which of the available places the user is actually present. If the focus of attention happens to be on multiple places at once (e.g. listening to a video call while typing in the command line), the journey temporarily forks into parallel cyber places. Consequently, analyzing individual journeys may sometimes require considering more than one sequence of movements at the same time.

### Diversity of journeying entities

Crime journeys have traditionally been framed from an offending perspective. Consistent with criminological tradition, our framework adopts this perspective. However, there is no reason to limit journeys to offenders alone; other journeying entities can also be considered. For example, from a convergence perspective of the minimal actors required for crime (Cohen & Felson, 1979), it may be useful to analyze the movements of *co-offenders* to study patterns of crime commission across different roles within a criminal network. It may also be valuable to examine the journeys undertaken by *victims* to identify patterns of risk exposure. Likewise, exploring the journeys of *guardians* could provide insights into how to design more effective online patrol routes. For offender, co-offender, victim, and guardian journeys, the journeying entities are human, and the components for their analysis outlined in “A framework to analyze cybercrime journeys” section apply to all.

However, in cybercrime—and especially with the rise of artificial intelligence (AI)—non-human entities are becoming increasingly relevant for crime analysis (see Campedelli, 2026). Journeys can now be automated and effectively outsourced to AI agents that carry them out, much like autonomous cars or drones do in physical environments. The online mobility of non-human journeying entities, such as AI agents and data itself, can also be examined through the topology of cyberspace and the cybercrime journeys framework, although with some adaptation. For example, instead of traversing the virtual layer, data journeys unfold through the physical infrastructure that supports information systems. There, nodes refer to infrastructure components such as routers and servers, while edges represent the cyber-physical connections between them, including both wired and wireless links. Data journeys are often unnoticed by users (though they may be aware of it) and remain largely outside their control, except in specific cases (e.g. when users intentionally reroute traffic through a VPN).

To remain analytically relevant though, journey components should be adapted to the specific type of journeying entity. For example, Table 4 presents some components specific to data journeys, along with their definitions, and the research questions they address. These components could be measured using data sources such as packet capture and intrusion detection systems.

**Table 4:** Example additional components of data journeys

Component	Definition	Question it answers
Data type	The type of data that is transmitted along the ‘route’. E.g. login credentials, HTML files	What type of data is transmitted?
Gateways	The intermediary access points through which data enters or exits a cyber place (e.g. port 443)	Where do the data pass through?
Traffic volume	The amount of data that is transmitted along the ‘route’. E.g. 10 MB between nodes ‘a’ and ‘b’	How much data is transmitted?
Protocols	The rules and procedures that determine how data ‘traffic’ is handled. E.g. TCP, HTTP, DNS	How is the data transmission handled?
Travel modes	The types of networks in which the data travels. E.g. the Internet, LAN, a Wi-Fi network	Which networks the data passes through?

Overall, this example illustrates the versatility of the framework in analyzing different journeying entities and adapting to the current challenges faced by criminology. Cybercrime journeys may be particularly well suited to addressing research questions and generating hypotheses within criminology and the social sciences, while also offering valuable insights for digital forensics, cybersecurity, and related areas of computer science. Note that the framework has been developed to support the analysis and investigation of any type of cybercrime and cybercriminal, but beyond analyzing illicit behavior, it could also be used to study human dynamics in cyberspace more broadly, including non-criminal activities.

### Compatibility with existing frameworks

The framework proposed here has the potential to transcend disciplinary boundaries, and complement existing analytic models, such as crime scripts in criminology (Cornish, 1993), and kill chains (Hutchins, Cloppert, & Amin, 2011), and tactics, techniques, and procedures (TTP) frameworks in cyber security (Strom et al., 2020). Like the (cyber)crime journeys, these three models share a common feature: they provide a sequential perspective on the crime commission process to identify points of intervention. Despite some overlap, they also exhibit important differences that make them more or less suitable for specific tasks, which highlights their complementarity.

Regarding the *timing of analysis*, the models enable the analysis of stages before, during, and after the crime, although they do not all emphasize the same stage. Criminological models tend to focus on the stages before and during the crime, whereas cyber security models primarily emphasize the execution phase. As a result, the former are particularly useful for examining the conditions that enable behavior, while the latter excel at disentangling the execution of the attack.

Another similarity is that these models allow for different *levels of analysis*: they can be applied to individual cases, but also aggregated to identify broader behavioral patterns. However, at the individual level, the value of crime scripts is more limited, as their typical organization into scenes already simplifies the crime commission process, leading to a notable loss of detail when applied to single cases. A similar issue arises with kill chains, which are structured into phases. By contrast, this limitation is less pronounced for TTP frameworks and journeys, as their more granular focus on actions and movements enables rich case analysis that is valuable on its own. The downside of this level of detail, however, is that it complicates aggregation, resulting in behavioral models that are complex to interpret.

Perhaps the most obvious difference between these models lies in their *units of analysis* (i.e. the basic element examined). Crime scripts focus on individual actions organized into scenes, kill chains on groups of actions within phases, TTP frameworks on (sub-)techniques within tactics, and journeys on movements within trips. This distinction makes journeys unique in their ability to engage with a broad range of environmental criminology theories, as their analysis is inherently spatiotemporal. Whereas crime scripts are typically aligned with a rational choice perspective in specific situations (Cornish, 1993; Leclerc, 2017), journeys allow for a richer examination of convergence patterns across a continuum of time and space, in line with the routine activity approach (Cohen & Felson, 1979) and crime pattern theory (Brantingham & Brantingham, 1993a).

### Limitations and future research directions

Although still in its early stages, we anticipate at least three limitations of this framework. First, it does not address the offline dimension of cybercrime. We acknowledge that cyber offenders are physically located somewhere and may also journey through geographical space before, during, and after committing cybercrime. Recent debates on the hybridization of crime (Roks et al., 2021) and the role of offline ties in cybercrime commission further develop these ideas (Loggen et al., 2026). Understanding how offline and online journeys intersect remains a topic for future research. Second, although we have aimed to formulate the framework in general terms, certain digital technologies—current or future—may require further adaptation. We leave this challenge for future research. Third, our framework adapts and extends existing criminological concepts rather than proposing an entirely new theoretical model for analyzing cybercrime journeys. While this approach leverages the strengths of environmental criminology, it also carries forward some of its potential limitations.

## Data Availability

The data analyzed in the empirical illustration of the framework presented here are publicly available via the Open Science Framework (OSF) at: 10.17605/OSF.IO/57HYV. The analysis code is also publicly available in the OSF at: 10.17605/OSF.IO/YX47N. A version of this manuscript has been uploaded as a preprint to SocArXiv Papers with 10.31235/osf.io/jybkv_v4.

## Footnotes

1 We recognize that this is a contested metaphor. For classic legal arguments cautioning against the “cyberspace as place” metaphor, see Lemley (2003); Olson (2005).

2 To be fully congruent with the geometry of crime, the term *cyber place* should be *cyber node*. However, given that the existing literature uses the term ‘cyber place’ to fit the criminology of place paradigm, and that ‘nodes’ have a specific meaning in computer networks, we opted to retain ‘cyber places’ to avoid confusion.

3 It may also be possible that the awareness space is influenced by sufficient information provided by third parties, but here we only consider the original definition of awareness space.

4 It would be possible to combine directed networks with stepwise timelines in a single three-dimensional visualisation (see Kapler & Wright, 2005), but for ease of interpretation we prefer to keep them separate.
